# A narrative review of the success of intramuscular gluteal injections and its impact in psychiatry

**DOI:** 10.1007/s42242-018-0018-x

**Published:** 2018-07-27

**Authors:** Erfan Soliman, Sarujan Ranjan, Tianyou Xu, Carol Gee, Aidan Harker, Alvaro Barrera, John Geddes

**Affiliations:** 1 Department of Engineering Sciences, Institute of Biomedical Engineering, Old Road Campus Research Building, University of Oxford, Oxford, OX3 7DQ UK; 2 Warneford Hospital, Oxford Health NHS Foundation Trust, Warneford Ln, Oxford, OX3 7JX UK; 3 Department of Psychiatry, University of Oxford, Warneford Hospital, Warneford Ln, Oxford, OX3 7JX UK

**Keywords:** Intramuscular injection, Gluteal, Subcutaneous, Ultrasound, Computed tomography, Antipsychotic

## Abstract

There are 12 billion injections given worldwide every year. For many injections, the intramuscular route is favoured over the subcutaneous route due to the increased vascularity of muscle tissue and the corresponding increase in the bioavailability of drugs when administered intramuscularly. This paper is a review of the variables that affect the success of intramuscular injections and the implications that these success rates have in psychiatry and general medicine. Studies have shown that the success rates of intended intramuscular injections vary between 32 and 52%, with the rest potentially resulting in inadvertent subcutaneous drug deposition. These rates are found to be even lower for certain at-risk populations, such as obese patients and those on antipsychotic medications. The variables associated with an increased risk of injection failure include female sex, obesity, site of injection, and subcutaneous fat depth. New guidelines and methods are needed in order to address this challenge and ensure that patients receive optimum care. Looking forward, the best way to improve the delivery of intramuscular injections worldwide is to develop uniform algorithms or innovative medical devices to confirm or guarantee successful delivery at the bedside.

## Introduction

Intramuscular (IM) gluteal injections are a commonly used method of administering medication within clinical medicine. Typical medications administered via this route include sedatives, hormonal therapies, long-acting antipsychotics, immunosuppressants, and vaccinations. This route is popular due to the rich vascular supply of the muscle and the benefits this provides in drug absorption.

Anatomically, the gluteal muscle is separated from the skin by varying amounts of subcutaneous tissue. Since this is not visible during the injection, the practitioner may penetrate and distribute the drug in the subcutaneous tissue accidently. The vascularity of subcutaneous tissue is reduced compared to muscle tissue, which may result in poor absorption and distribution of the drug [1].

Unsuccessful IM injections can have a significant impact on the care of patients in both general medicine and psychiatry. For example, insufficient doses of drugs such as vaccines can have a negative effect on the health of the population, due to lower than expected immunity levels that may result [2]. In psychiatry, poor absorption of long-acting antipsychotic medications that lead to subtherapeutic drug levels in the body could result in relapse of symptoms of psychosis and potentially admission to hospital [3]. If the accuracy of intramuscular injections could be improved, not only would there be a possible reduction in relapse rates for patients with schizophrenia, but drug dosing could be done more effectively for these patients, which in turn would lead to fewer side effects and an improved quality of life. These changes would also have a significant impact on healthcare costs.

A number of studies have investigated the topic of IM injections and have identified significantly low rates of success [1, 4, 5]. Some studies have researched placement of the injection via computed tomography (CT) [1, 4]. Other studies used ultrasound or CT to indirectly compare the thickness of subcutaneous tissue to the length of the needles used, in order to predict rates of successful injection [6, 7]. This review paper summarises the current literature on this topic, highlights the relevance to psychiatry, and offers an outlook on future developments.

## Success rates of intramuscular injections

Several studies over recent years have suggested that failure rates of IM injections (those that did not reach the intramuscular space) are significantly high.

Boyd et al. [4] found an IM injection success rate of only 52% in patients (*n* = 115) undergoing treatment for carcinoid syndrome, evaluated by CT. The most common reason for misplaced injections was insufficient injection depth, which accounted for 36% of these cases. Other reasons included non-ideal injection site selection. Interestingly, the rate of success increased to 75% after additional training was provided to the nurses.

Garris et al. [5] examined octreotide long-acting injections, (LAIs) intended for gluteal muscle administration, and found that only 58% of all injections (*n* = 251) were deemed successful, evaluated by CT. Furthermore, it was found in the study that females had a lower body mass index (BMI) on average; however, due to greater skin-to-muscle depth at the optimal injection site, they experienced lower rates of success compared to males. The study also gathered experience levels (self-reported by the nurses) and found that experience affected success rates of gluteal IM injections to a greater degree in female patients compared to male patients.

Another study by Chan et al. [1] found that only 32% of intended IM injections reached the appropriate site, evaluated by CT in a heterogeneous patient population (*n* = 50). In this study, each patient received an IM injection of their prescribed medication along with 1 mL of air into the upper outer quadrant of the buttock prior to CT. These images were then analysed to determine the position of the injected air bubble in order to determine whether it was IM or subcutaneous. Crucially, when analysed by sex, 56% of males (*n* = 14/25) had IM injections, while the rate was significantly lower in females: only 8% (*n* = 2/25).

Post-injection success rates have also been evaluated by ultrasound and indirect measurements comparing thickness of subcutaneous tissue and length of needle used. For example, Zaybak et al. [6] acquired ultrasound measurements of the dorsogluteal and ventrogluteal sites where the probe was held at a 90° angle to the plane of the injection site to determine thickness of the subcutaneous tissue. Measurements were acquired in patients (*n* = 119) with BMI greater than 25 kg/m^2^. Their results suggested that, when using a 1.5 in needle, injections administered at the dorsogluteal site would not reach the muscle in 98% of women and 37% of men. Similarly, at the ventrogluteal site, injections would be unsuccessful in 97% of women and 57% of men [6].

There are also retrospective studies where subcutaneous fat depth was measured over the gluteus muscle using either CT or magnetic resonance imaging (MRI) to determine whether the length of needles being used was sufficient to reach the muscle. One study used automated CT calipers to determine the minimum distance between the surface of the skin and the nearest edge of muscle at the ventrogluteal and dorsogluteal injection sites in 100 patients [7]. Results suggested that in the posterior gluteal site, large needles (35 mm) will fail to reach muscle in 43% of the patients, and small needles (25 mm) will fail in 72% of the patients. Furthermore, the analysis found that the intramuscular site was likely to be deeper for females.

Another study acquired CT scans (*n* = 298) and measured the thickness of the subcutaneous tissue [8]. The average gluteal fat thickness for female subjects (*n* = 150) was found to be 33.2 mm, whereas the average for male subjects (*n* = 148) was 23.1 mm. Their results suggested that a 37-mm needle, allowing for adequate penetration of the gluteal muscle, would not have reached IM depth in 54.7% of female subjects (81 of 148), in 14% of male subjects (21 of 150), or on average in 34.2% of the total sample.

A more recent 2016 retrospective study measured the thickness of subcutaneous tissue using MRIs of the pelvis (*n* = 350, 224 women, 126 men) [9]. Their results suggested that a BMI of 30 in women and 35 in men seems to be upper limits for successful ventrogluteal IM injections with 3.75-cm hypodermic needles, and that the predicted failure rate of ventrogluteal IM delivery would be 71% in women with BMI > 30, and 60% in men with BMI > 35 (Table 1).

**Table 1:** Summary of intramuscular injection success rates from four prospective studies in which outcomes were directly measured using CT or ultrasound. (Reproduced with permission from [1, 4–6])

Paper	Year	Patient population	Success rate	Modality	Select notes
[3]	2006	*n* = 50 (25 males and 25 females)	32% (56% in males, 8% in females)	CT	Patient received an IM injection of their prescribed medication along with 1 mL of air. CT images were analysed to determine the position of the injected air bubble and to assess whether the injection was successful
[4]	2007	*n* = 119 with BMI > 24.9 kg/m^2^ (60 males and 59 females)	2–63%	Ultrasound	Using a 38-mm (1.5 in) needle, injections at the dorsogluteal site in 98% of women and 37% of men would not reach the muscle
[2]	2010	*n* = 251 intended IM injections of octreotide for control of carcinoid syndrome	58%	CT	Successful IM injection rate was lower in females than in males (42 vs 77%) as evaluated by CT
[1]	2013	*n* = 328 intended IM injections of octreotide for control of carcinoid syndrome	52%	CT	After further training to nurses, success rate increased from 52 to 75% (66–75% in males and 38–75% in females)

## Factors that affect injection success

The success of an intramuscular injection is therefore dependant on multiple variables. One must take into account the patient’s anatomy and how it informs the selected site of injection, the technique used by the nurse, the amount of subcutaneous tissue and muscle in the gluteal area, and the length of the needle chosen. Unfortunately for nurse practitioners, there are no clear guidelines available in the literature for the gold standard technique and therefore decisions are usually made based on experience and perceptions regarding historical practice. Interestingly, Boyd and colleagues have shown that more experience, in terms of number of years of nursing, did not improve the success rates of intramuscular injections. However, there is a significant increase in success rates for nurses who give intramuscular injections more frequently and for those who felt more comfortable giving the injection [4].

### Anatomy

**Fig. 1:**
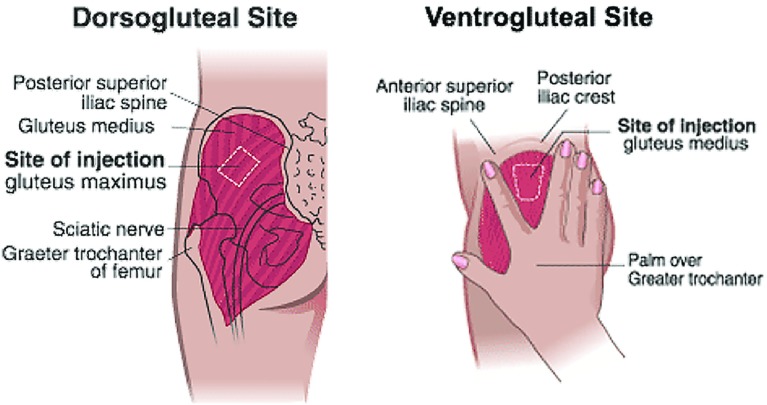
An outline of the dorsogluteal and ventrogluteal injection sites

The first variable to address when performing an IM injection is choosing the site of medication delivery. There are five sites where a IM injection can be administered: dorsogluteal, ventrogluteal, vastus lateralis, rectus femoris and deltoid [10]. For the purpose of this review, we have focussed on gluteal injection sites only (dorsogluteal and ventrogluteal). Traditionally, the choice of site for gluteal injections has been based upon nurse preference and confidence rather than evidence gleaned from primary research that ensures methodological and ethical standards have been applied. In making this decision, factors such as the drug used, the injection volume, and patient preference should be taken into account. Historically, nurses would give the intramuscular injection in the dorsogluteal area which is located in the ‘upper outer quadrant’ of the gluteal muscle [11]. However, evidence-based literature has disputed the use of the dorsogluteal area in favour of the ventrogluteal area, with regard to safety [12]. The dorsogluteal area is close to the sciatic nerve and gluteal artery; it is therefore possible to injure these vessels with an injection into this area [13]. In addition, it has been shown that the dorsogluteal area has greater amounts of subcutaneous fat compared to the ventrogluteal area, meaning that an injection in this site has a greater chance of failure [14] (Fig. 1).

### Technique

Once the site of injection has been determined, the patient should be positioned such that the target muscle is relaxed in order to avoid discomfort [15]. The next consideration is the method of injection. It has been recommended that the needle is injected at a 90° angle as this gives the best possible chance for intramuscular penetration [16]. In a study undertaken by Marshall et al. [17], it was suggested that although an angle of 60°–90° should work for most patients, an angle of 60° may cause inadvertent subcutaneous injections in obese patients. The speed of injection is another important variable. Quick insertion of the needle will result in reduced pain for the patient. It has also been recommended that the plunger is depressed at a rate of approximately 0.1 mL/s to avoid patient discomfort [18].

The most common technique used by practitioners is the z-track method. This involves the practitioner using their non-injecting hand to laterally displace the skin and subcutaneous tissue prior to injection [19]. In a recent study by Yilmaz et al. [20], it was shown that the z-track technique reduces the leakage of the intramuscular drug into the subcutaneous tissue. Once again, there is no clear guidance in the literature about which technique is appropriate. In practice, some nurses also use their non-injecting hand to ‘bunch’, ‘stretch’ or ‘depress’ the skin. A study by Palma and colleagues found that in obese women, the injection should be given without bunching of the skin in order to reduce the risk of subcutaneous misplacement [21]. A study by Boyd et al. [4] found that depression of the skin had a higher intramuscular success rate than bunching or stretching.

In psychiatry, further considerations need to be taken into account regarding technique and site of injection. The ventrogluteal site is taught in clinical practice and within nurse training as the site of choice; however, due to concerns within mental health practice regarding the amount of physical contact required to landmark safely, this injection site is not routinely used. Instead, mental health nurses tend to move to the upper outer quadrant of the dorsogluteal area (which itself is in the upper outer quadrant of the gluteal muscle). If landmarked correctly, this approach avoids the sciatic nerve and artery. The technique of mental health nurses is otherwise the same as that used in general medicine, and guidance is gleaned from the NHS clinical skills website [22]. All IM injections are given at an angle of 90°, all are z-tracked, and all are administered at a pace of 10 s per mL (and also left a further 10 s before needle withdrawal to ensure absorbency).

**Fig. 2:**
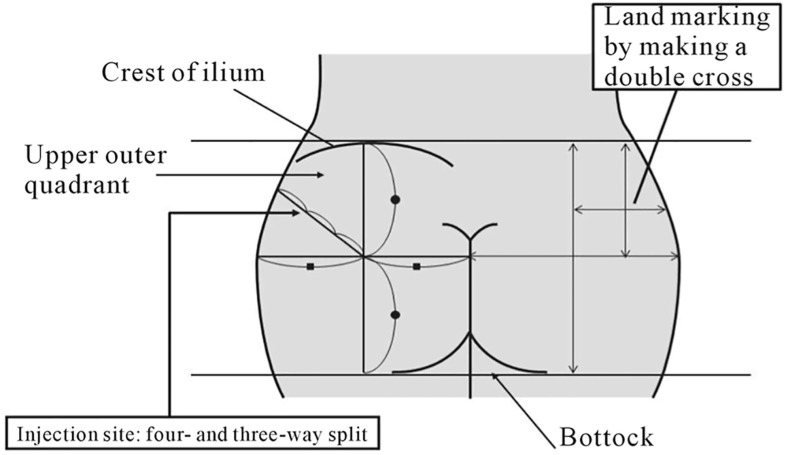
Summary image of the techniques used to landmark the various regions on the buttock prior to intramuscular injections. (Reproduced with permission from [22])

Traditional practice amongst mental health nurses involves the use of two sites: dorsogluteal and deltoid, both of which require minimal physical contact to landmark and can be easily accessed with the patient in a lying or standing position. Some patients receiving an intramuscular LAI may at times not be cooperative or compliant. This may cause practitioners to administer the injection too quickly without taking the required time to measure out anatomical landmarks. This may also lead to poor technique and may play a role in failed IM antipsychotic injections. An important consideration is also patient choice. Some patients do not like receiving gluteal injections and must therefore be presented with the appropriate resources required to make an informed decision (Fig. 2).

### Subcutaneous fat, BMI, and gender

**Fig. 3:**
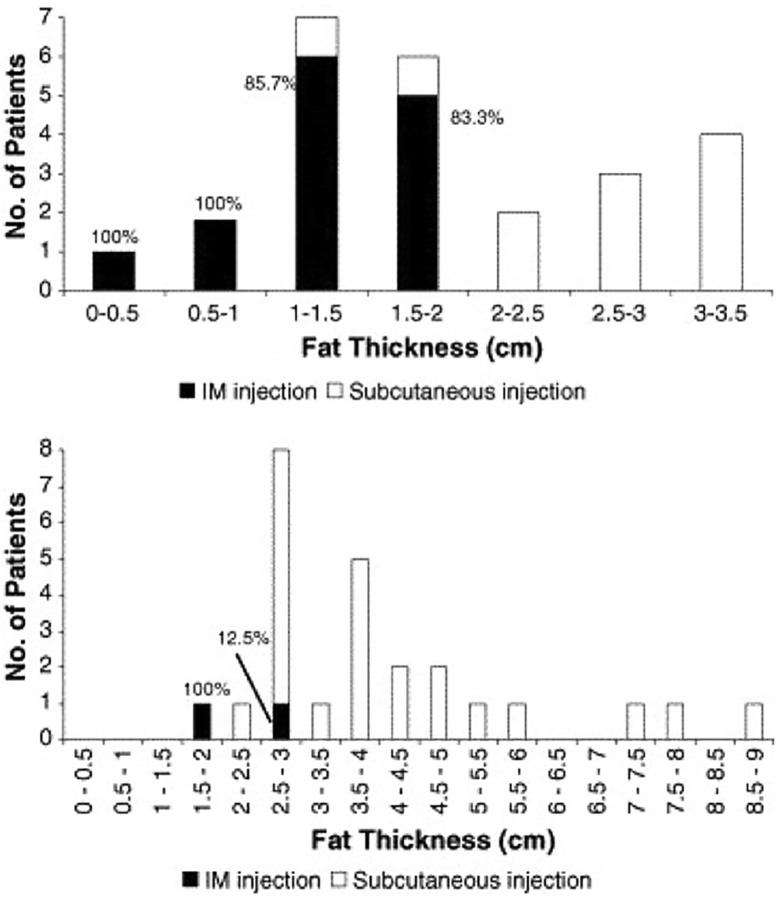
Comparison of subcutaneous fat thickness and successful intramuscular injections in males (top) and females (bottom). (Reproduced with permission from [1])

High subcutaneous tissue (SCT) thickness has been shown to be the main predictor of unsuccessful IM injections. Chan et al. [1] found that, overall, out of those who received a successful IM injection, the mean SCT thickness was 13.6 mm (range: 3.7–25.4 mm), while those who received an unsuccessful injection had a mean SCT thickness of 36.2 mm (range: 14.0–87.0 mm). Despite these results, studies exploring the correlation between BMI and gluteal SCT thickness report varied findings, so drawing conclusions about IM injection success from an individual’s BMI is not always possible. For example, the study found that BMIs of under 20 and over 30 had a significant impact on the probability of IM injection success (positive and negative, respectively); however, the difference in success rates amongst those with BMIs in the low 20s and those with BMIs in the high 20s remained inconclusive [1] (Fig. 3).

**Fig. 4:**
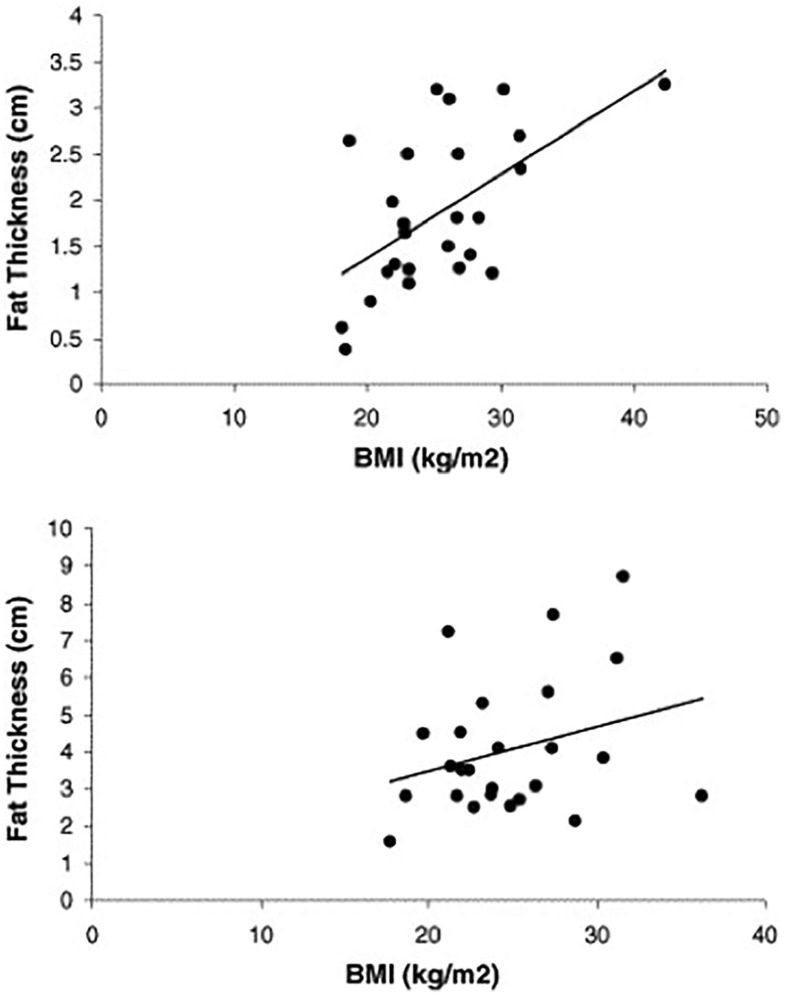
Relationship between BMI and subcutaneous tissue thickness in males (top) (Spearman’s *r* = 0.51, *P* < 0.01) and females (bottom) (Spearman’s *r* = 0.19, *P* = 0.37). (Reproduced with permission from [1])

However, there is evidence to suggest that patient sex can determine whether or not a correlation exists between gluteal SCT thickness and BMI. Even though it might be expected that SCT thickness would always increase with BMI, the findings indicated that this was only true in males (Spearman’s *r* = 0.51, *P* < 0.01) and not for females (Spearman’s *r* = 0.19, *P* = 0.37) [1] (Fig. 4).

Similarly, another analysis reported mean SCT thicknesses of 34.5, 40.2, and 51.4 mm for overweight (BMI 25–29.9), obese (BMI 30–34.5), and extremely obese (BMI > 35) adults at the dorsogluteal site, and 38.2, 43.1, and 53.8 mm at the ventrogluteal site, respectively [6]. At the dorsogluteal site, differences amongst BMI groups were again only significant in men (*F* = 4.609, *P* < 0.05), and not in women (*F* = 0.708, *P* > 0.05). At the ventrogluteal site, differences amongst BMI groups were not significant in men nor women (*F* = 2.982 in men and *F* = 2.210 in women, both *P* > 0.05).

Though the correlation between SCT thickness and BMI is not always straightforward, an analysis that included measurements of the distance from the epidermis to the under-fascia (DEUF) of the gluteus maximus, the DEUF of the gluteus medius, as well as the distance from epidermis to the iliac bone (DEI) found that statistically significant correlations could be seen between DEI and BMI data [23]. In fact, the study concluded that DEI might be approximately proportional to BMI. Because DEI measurements include the thicknesses of both SCT and muscle, there is some scope for using the relationship to inform IM injection technique.

Furthermore, a cross-sectional study of muscle and SCT thickness found that, overall, dorsogluteal SCT, muscle, and total tissue thicknesses were significantly greater than ventrogluteal thicknesses (*P* < 0.001). SCT thickness at both sites was found to be significantly greater in females than in males (*P* < 0.001), but there was no significant gender difference in total tissue or muscle thickness at either site. In developing an algorithm for injection site selection, it was found that weight and BMI for males and females, waist and hip circumference for females, and the distance between iliac tubercle and anterior superior iliac spine for males can be used to predict accuracy and safety of IM injection [24]. These findings can inform new approaches to IM injections, as well as the design of any devices that are manufactured for ensuring injection success.

Given the rampant rates of obesity around the globe [25], addressing this challenge is a matter of priority. This is especially the case in the field of psychiatry for patients suffering from severe mental illnesses (SMIs), who are at an even greater risk for obesity than the general population. These risks are not only associated with medical complications such as diabetes and cardiovascular disorders [26, 27], but also the potential worsening of the patient’s mental illness due to treatment noncompliance or reduced quality of life [28]. Mortality rates amongst the mentally ill are also much higher than in the general population, such that those suffering from an SMI are expected to live 25 years less on average by some estimates [29, 30].

Antipsychotic medications are often fundamental to the treatment plans of individuals with SMIs; however, these drugs themselves provide an added risk of weight gain, obesity, and cardiovascular disease [26–28]. Furthermore, attempts to treat or prevent the cardiometabolic side effects of these drugs are not very effective [31].

In the case of antipsychotic injections, the mentally ill population is therefore particularly at risk of inadvertent subcutaneous drug deposition. Cognizance regarding the clinical consequences of drug-induced obesity has been on the rise, for antipsychotic medications in particular [26–28, 31, 32]; however, recent findings indicate that the extent to which this problem impacts patient populations receiving chronic treatment is invariably underestimated. BMI increases of up to one unit (kg/m^2^) are observed within 12 weeks of treatment with the commonly prescribed antipsychotic, risperidone [32]. Moreover, a patient’s first exposure to antipsychotic medication has an even larger effect, with BMI gains of 1.5 kg/m^2^ observed after 12 weeks on risperidone in the Comparison of Atypicals for First Episode (CAFE) trial for adults experiencing an episode of schizophrenia for the first time [8]. This finding is nearly three times higher than that reported in the initial phase of the Clinical Antipsychotic Trials in Intervention Effectiveness (CATIE) study [32], which is the largest antipsychotic comparison study to date. The weight gain associated with these drugs is clearly problematic, as both drug manufacturers and NICE guidelines state that these medications should only be administered intramuscularly; absorption rate, blood serum concentration, and dopamine receptor occupancy can be affected in the case of an unsuccessful injection. The risk is even higher in the case of long-acting injections (LAIs), which are often given in the form of an oil depot, with low solubility, to release the drug over a longer period of time. Because of the lowered frequency of these injections (monthly or even three-monthly), it is increasingly important that they be delivered to the right place, within muscle tissue. To date, there have been no studies evaluating the risk of relapse associated with failed IM injections for patients receiving antipsychotic LAIs. This is a big potential problem given that SMIs like schizophrenia and bipolar disorder are extremely costly for healthcare systems around the world, over £2 billion for schizophrenia [33] and over £342 million for bipolar disorder [34] in the UK alone.

### Needle length and choice

IM injections are defined as injections in which the needle tip pierces the muscle by at least 5 mm [35]. Furthermore, historical practice recommends that approximately 2–3 mm of the needle length is left outside the skin to allow for removal should the needle break. As such, needles used for IM injections are typically 25–38 mm (1–1.5 in) long and 19–22 gauge. Needle gauge is the measure of the thickness of the needle and ranges from 7 gauge (the largest) to 33 (the smallest) on the Stubs scale [36]. 21-gauge needles are most commonly used for IM injections.

Guidelines for site/location and needle size (length and gauge) for adult, paediatric, and infant IM injections exist [37]. For example, in adult populations, recommended needle length ranges from 25 to 38 mm and can go up to 76 mm (3 in) for large, obese adults. Recommended needle gauge ranges from 19 to 25. Typically, the administrator of the injection makes the decision about needle size. This decision is based on weight and BMI of the patient, site and route of delivery, viscosity of the medication, and the amount of medication to be given. However, where needles are supplied with an injection by the manufacturer, only those needles should be used. The following are needle size recommendations for common antipsychotic IM LAIs from *Guidance on the Administration to Adults of Oil*-*based Depot and other Long*-*Acting Intramuscular Antipsychotic Injections, 5th Edition* [10].

*Aripiprazole* Only needles recommended and supplied in the dose pack may be used for the injection of aripiprazole LAIs. The gluteal administration of the aripiprazole LAI calls for a 38-mm, 22-gauge hypodermic safety needle; for obese patients with a BMI > 28 kg/m^2^, a 50-mm, 21-gauge hypodermic safety needle should be used. For deltoid site administration, the recommended needle is a 25-mm, 23-gauge hypodermic safety needle and for obese patients, a 38-mm, 22-gauge hypodermic safety needle [10].

*Olanzapine* Only the Needle-Pro safety needles supplied in the dose pack may be used for administering olanzapine LAIs. For obese patients with a BMI > 30 kg/m^2^, the 50-mm needle is recommended. Otherwise, the 38-mm safety needle should be used [10].

*Paliperidone palmitate* Only the needles supplied in the dose pack should be used. The 22-gauge 38-mm safety needle should be used for dorsogluteal and deltoid injections in patients over 90 kg. The 23-gauge 25-mm safety needle should be used only for deltoid injections in patients under 90 kg [10].

*Risperidone* Only needles supplied in the pack by the manufacturer may be used for administering risperidone LAIs. The packs contain two needles and both are fitted with a Needle-Pro safety device. The crucial feature in each case is the needle bore width. These needles have thin walls which result in a larger bore than standard needles, which allows the risperidone liquid suspension to flow more easily through the needle. The 50-mm needle is to be used for gluteal site injections, and the 25-mm needle is to be used for deltoid site injections [10].

Gauge, bore width, and needle length recommendations vary across the four common antipsychotic medications as described above. While bore width should vary depending on the different physical properties of the medication, inconsistency in needle length recommendations suggests a more fundamental issue. In the administration of paliperidone palmitate, the largest needle length recommended is 38 mm, which is for use on patients over 90 kg. In contrast, needle length in aripiprazole administration is determined by the patients’ BMI, with 50 mm being the longest needle available. These contrasts highlight the inconsistency in medical practice with regard to IM injections.

## Consequences of unsuccessful injections and effect on medical practice

Though requirements for intramuscular injections can vary, a 32-mm (1.25 in) needle is often used in standard cases [35]. When poor technique or a thick SCT layer (> 25 mm) results in inadvertent administration of the medication within subcutaneous tissue, it can result in a variety of complications such as localised tissue damage, reduced drug efficacy, and a slower rate of drug absorption [11, 38, 39]. Granulomas occur from subcutaneous IM injections (intralipomatous) resulting in fat necrosis and dystrophic calcification [40]. As discussed thus far in this article, inadvertent subcutaneous injections are a common occurrence, and some studies have found that most injections that are thought to be IM are in fact subcutaneous [6].

Furthermore, on infrequent occasions, the needle can come into contact with bone, or even penetrate osseous tissue in rare cases when there is inadequate SCT and/or muscle thickness. This can result discomfort in the form of a bony contusion, or in more serious cases osteonecrosis [41]. For standard needles, 35 mm is the minimum tissue thickness required to allow for a penetration depth of 30 mm and a margin of safety [24].

Outside of mental health and psychiatry, intramuscular injections are used for a variety of different clinical purposes. There are 12 billion injections given per year worldwide [19]. Examples of medications which can be given intramuscularly include hormonal therapies, vaccinations, sedatives, adrenaline, and immunosuppressants. Poor absorption of these medications can lead to subtherapeutic levels of the drug in the body and a poor clinical outcome.

For example, most vaccines are given via the intramuscular route. Studies have suggested that if medication is given subcutaneously instead of intramuscularly, this can lead to poor processing of the antigen and vaccine failure. This includes vaccines such as hepatitis B and influenza [42]. Failure of the influenza vaccine in particular can pose a significant health risk to vulnerable groups such as the elderly and patients with complex medical problems [43].

Some hormonal drugs are delivered via the intramuscular route. hCG has two formulations: originally urinary hCG (u-hCG), which is given intramuscularly, and more recently recombinant hCG (r-hCG), which is given subcutaneously [44]. A study conducted by Chan and colleagues found that when directly comparing the bioavailability of 10,000 IU of Pregnyl, the bioavailability of the drug was much greater intramuscularly. The study also found that the bioavailability of hCG was significantly reduced in obese compared to non-obese women [45]. A more recent study found that after subcutaneous injection of r-hCG, bioavailability of the drug was much lower in obese patients. However, following intramuscular injection of u-hCG, the bioavailability of the drug was similar in both obese and non-obese patients [44]. This is an important consideration, when appreciating that accidental subcutaneous injection and poor bioavailability after such an injection could result in failure of therapy.

Intramuscular injections can also play a fundamental role in medical emergencies. In a scenario where someone is suffering from anaphylaxis, it is imperative that the patient receives an immediate dose of adrenaline intramuscularly. Studies undertaken by Simmons and colleagues show that intramuscular delivery leads to the patient receiving higher peak levels of adrenaline in a shorter timeframe than is the case with subcutaneous delivery. It is believed that this rapid peak could be essential to the survival of the patient via reversal of anaphylaxis [29]. Unfortunately, a study undertaken by Stecher et al. [46] in children between the age of 1–12 found that 30% of them had a skin-to-muscle distance that an adrenaline autoinjector needle would not penetrate. Recent studies in adults have found that the autoinjectors used do not reach the muscle in many patients, especially in women [47, 48].

It is clear that in many medical fields, ensuring intramuscular delivery of drugs can play a large role in effective patient care. This can have not only a significant impact on patient quality of life but on healthcare costs in general.

## Guaranteeing injection success

Innovative design and medical device technologies can help address this clinical need. In the following section, we will discuss the state of current solutions as well as identify concepts for potential future development.

### Risperidone subcutaneous implant

Risperidone is a second-generation antipsychotic approved in the USA and the European Union for the treatment of schizophrenia, traditionally formulated as daily oral tablets or as bi-weekly intramuscular depot injections. Although long-acting injectable antipsychotics improve treatment compliance, these agents are still associated with certain disadvantages, such as slow dose titration, pain at the injection site, requirement for periodic drug administration by a healthcare professional, and inability to withdraw or reverse the drug in case of emergency [49]. A new risperidone formulation for subcutaneous implantation was purchased by Braeburn Pharmaceuticals Inc from Endo Pharmaceuticals and reformulated as an implant. This formulation is designed to be implanted in the upper arm and to release the active drug at a constant rate for 6 months. Advantages of the implant include a significantly longer treatment effect after each administration (6 months), ability to quickly remove the implant for urgent discontinuation, improved convenience due to fewer clinic visits, potentially increased patient compliance due to decreased frequency of administration compared with oral tablets and injections [50], reduced stigma associated with regular medication administration, and potentially decreased healthcare costs resulting from reduced clinic visits.

### ATRIGEL delivery system

In 2017, Indivior announced positive results from its Phase 3 open-label, long-term safety trial of RBP-7000 risperidone monthly depot for the treatment of schizophrenia [51]. RBP-7000 is a novel sustained-release product using the ATRIGEL delivery system for the subcutaneous administration of risperidone once every month [52]. The drug mixture is injected subcutaneously as a liquid into the patient’s abdomen, where it subsequently solidifies, resulting in the prolonged release of risperidone for 1 month before it eventually biodegrades. If such a system can be demonstrated to be as clinically efficacious as current treatments, it may serve as an attractive replacement as it both lengthens the time between risperidone administrations and can be delivered subcutaneously, overcoming the issue of missed intramuscular injections altogether.

### Needleless injection

The two example technologies above are at a mature stage and may realistically begin exercising their impact in the near future. Still, other novel technologies exist that may address the medication delivery problem through other means. For example, MIT spinout Portal Instruments landed a commercialisation deal in 2017 for a smart, needle-free injection device based on technology that could reduce the pain and anxiety associated with needle injections, shorten administration time, and improve patient adherence [53]. The technology was first developed in 2012, taking on the form of a jet-injection device that delivers a rapid, high-pressure stream of medicine, as thin as a strand of hair, through the skin in adjustable dosages, causing little to no pain to the subject. The device could be adjusted to accommodate different medications. Conceptually, such a technology could even be developed to allow for intramuscular antipsychotic injections or for subcutaneous injections of antipsychotic medications of future generations.

### Novel designs and 3D printing

Biocompatible materials can be used to create functional tools for applications in both tissue engineering and drug delivery. For example, 3D printing technology has been used to manufacture innovative needle designs that may have an important role to play in these fields. A recent paper presented new designs of bio-inspired needles to be used for injections [54]. Insect stingers have long been known to easily penetrate soft tissues. Bio-inspired needles mimicking the barbs in a honeybee stinger were developed for a smaller insertion force, which can provide a less invasive procedure. Decreasing the insertion force will decrease the tissue deformation, which can alleviate discomfort for patients. Multiple scales and variations of the barbed needles were designed and used to explore the size-scale effect on the insertion force [54]. Such designs may be useful in the development of longer needles, which may be necessary for use in some patients in order to reach the muscle layer in an IM injection. However, longer needles are typically thicker so as to preserve their tensile strength, and the increased thickness of the needle may cause greater pain and discomfort for patients upon injection. Novel bio-inspired designs may allow manufacturers to create needles of sufficient length and tensile strength while reducing pain upon delivery. 3D printing technology may play a critical role in the development of such needles.

3D printing has also been used to produce high aspect ratio polymer resist microneedles on a silicon substrate. These needles are then made functional by using an iron coating, which gives them an isotropic magnetic behaviour. Two-photon polymerisation lithography was used for printing cylindrical, pyramidal, and conical needles—highlighting the power of 3D printing technology in producing electromagnetically functional microneedles that benefit from flexibility in geometry and shape. The results showed that the iron-coated needle arrays had high biocompatibility, opening the door for potential applications in tissue engineering and drug delivery [55].

## Conclusion

It is clear from the literature that there remains great uncertainty around the success rates of IM injections in current clinical practice. Seeing as there are studies that demonstrate success rates varying from 32 to 52%, there is a clear need for improvement. With the combination of rising obesity levels and lack of formal guidelines, the risk of a failed injection extends to an increasingly large patient population, potentially affecting their quality of life and even safety.

As with any blind procedure, the practitioner cannot be sure that the injection is ever truly intramuscular. All of the variables affecting IM injections should be taken into consideration in order to avoid compounding risk; these variables include: female sex, obesity, site of injection, and subcutaneous tissue thickness. Therefore, the only way to improve the delivery of IM injections worldwide is to develop strict guidelines, algorithms, or innovative medical devices to confirm or guarantee intramuscular delivery at the bedside. There is increasing possibility of using 3D printing technology to provide such a solution. It is also encouraging that new and reliable methods of drug delivery are being developed, such as subcutaneous implants and needleless injection devices. This may reduce the reliance on traditional approaches to IM injections in the future.
